# Supplementary material for: Does how individuals handle social situations exacerbate the relationship between physique anxiety and food addiction? The role of emotional expressive suppression and social avoidance and distress
Source: PeerJ. 2024 Aug 16;12:e17910. doi: 10.7717/peerj.17910 (PMC11332389; doi:10.7717/peerj.17910)
Supplement: Supplemental Information 2 [file peerj-12-17910-s002.docx]

Dear Editor and Peer Reviewer:

In accordance with the requirements of the journal and for your convenience in reviewing the manuscript, we have provided you with the codes for converting the categorized data into their respective factors.

1.C-mYFAS2.0

2. ES

Its a 7-point Likert scale with scores ranging from 1 to 7 indicating “Strongly Disagree” to “Strongly Agree,” respectively.

3. SPA

All items were scored on a 5-point Likert scale, with scores ranging from 1 to 5 indicating “strongly disagree” to “strongly agree”, and 1, 3, 7, 11, 13, and 14 of the 15 items were reverse scored. The total score ranges from 15 to 75.

4. SAD

All items in the scale are assessed by “yes” or “no”, with a score of 1 if the answer is “yes “and 0 if the answer is “no”, and items 1,3,4,6,7,9,12,15,17,19,22,25,27,28 are reverse scored. The total score ranges from 0 to 28.
