# Supplementary material for: Does how individuals handle social situations exacerbate the relationship between physique anxiety and food addiction? The role of emotional expressive suppression and social avoidance and distress
Source: PeerJ. 2024 Aug 16;12:e17910. doi: 10.7717/peerj.17910 (PMC11332389; doi:10.7717/peerj.17910)
Supplement: Supplemental Information 3 [file peerj-12-17910-s003.docx]

**Authorization on measuring tools**

Dear Editor:

Thank you for proposing the revision. The four scales used in this study are authorized by the authors for public use in the Chinese population, as evidenced by the following:

1. SPAS:SPAS was developed by Xu, and Xu proposed that the scale could be used in China.

2.ESS

Specifically, the Emotion Regulation Strategies Scale is an author-authorized scale that can be widely used globally, and we used the Chinese version of the ERSS, which the original authors have authorized for use in Chinese populations.

3.SAD

The SAD scale was developed as early as 1969, and it is also an author-authorized and widely used scale worldwide. We used the Chinese version of the SAD, which has been authorized by the original author for use in the Chinese population.

4.C-mYFAS2.0

We used the Chinese version of the C-mYFAS2.0. This scale has been authorized by the original author and should be further validated.
